# Case report: an aortic aneurysm as cause of pseudoachalasia

**DOI:** 10.1186/s12876-020-01198-y

**Published:** 2020-03-06

**Authors:** Marian Dejaeger, Maud Lormans, Eddy Dejaeger, Katleen Fagard

**Affiliations:** 1 Department of Geriatric Medicine, University Hospitals Leuven, Leuven, Belgium; 2 Department of Public Health and Primary Care, Laboratory of Gerontology and Geriatrics, KU Leuven, Leuven, Belgium; 3 Faculty of Medicine, KU Leuven, Leuven, Belgium

**Keywords:** Ageing, Aortic aneurysm, Pseudoachalasia, Dysphagia aortica

## Abstract

**Background:**

Pseudoachalasia is a rare disorder which has clinical, radiographic, and manometric findings that are often indistinguishable from primary achalasia. It is usually associated with malignancy. Few reports describe vascular compression as a cause of pseudoachalasia.

**Case presentation:**

Here we present a case of a 84-year-old woman with anorexia, dysphagia and unintentional weight loss initially diagnosed as achalasia. Upon further investigation a rare cause of pseudoachalasia due to vascular compression of the esophagus was found. It could have been overlooked due to the fact that the initial work-out with a barium swallow, manometry and endoscopy was suggestive for primary achalasia.

**Conclusion:**

Particularly in older patients with a manometric diagnosis of achalasia, additional investigation to rule out pseudoachalasia is warranted. Although malignant involvement of the esophagus is the most common cause of pseudoachalasia, benign origins have also been described.

## Background

Dysphagia is defined as the sensation of difficulty or discomfort in swallowing. It is considered a warning symptom for potentially serious underlying causes, especially in the elderly population. Among older persons, dysphagia is a common symptom, with prevalence rates between 30 and 40% in independently living older people and more than 50% in institutionalized older patients [1]. Dysphagia symptoms can present as high or low dysphagia. High or oropharyngeal dysphagia is mostly caused by hypocontractility of the hypopharynx or by incomplete relaxation of the upper esophageal sphincter. Low or esophageal dysphagia is often due to motor dysfunction of the esophagus (insufficient peristalsis or uncoordinated contractions) or incomplete relaxation of the lower esophageal sphincter (LES) muscle, but can also be caused by gastro-esophageal reflux disease or by more rare causes of esophageal stricture [2]. Mechanical obstruction of the esophagus, either caused by intra-luminal or external compression, is a less frequent cause of low dysphagia.

Achalasia can be defined as incomplete relaxation of the LES and insufficient peristalsis of the esophagus. In the Chicago classification, a diagnosis of achalasia is based on an elevated median integrated relaxation pressure (> 15 mmHg) in combination with failed peristalsis or spasm [3]. Pseudoachalasia is indistinguishable from achalasia based on manometry. The most common etiology of pseudoachalasia is malignant submucosal involvement of the esophagus. However, other causes, such as previous local surgery or external compression of the esophagus, occur as well. The rare condition in which external compression of the esophagus secondary to an aortic aneurysm or a dissecting or tortuous aorta leads to dysphagia is called dysphagia aortica and was first described by Pape in 1932 [4].

Herein, we present a case of an elderly female with an initial diagnosis of achalasia, but where additional investigations led to the diagnosis of pseudoachalasia due to an aortic aneurysm.

## Case presentation

**Fig. 1:**
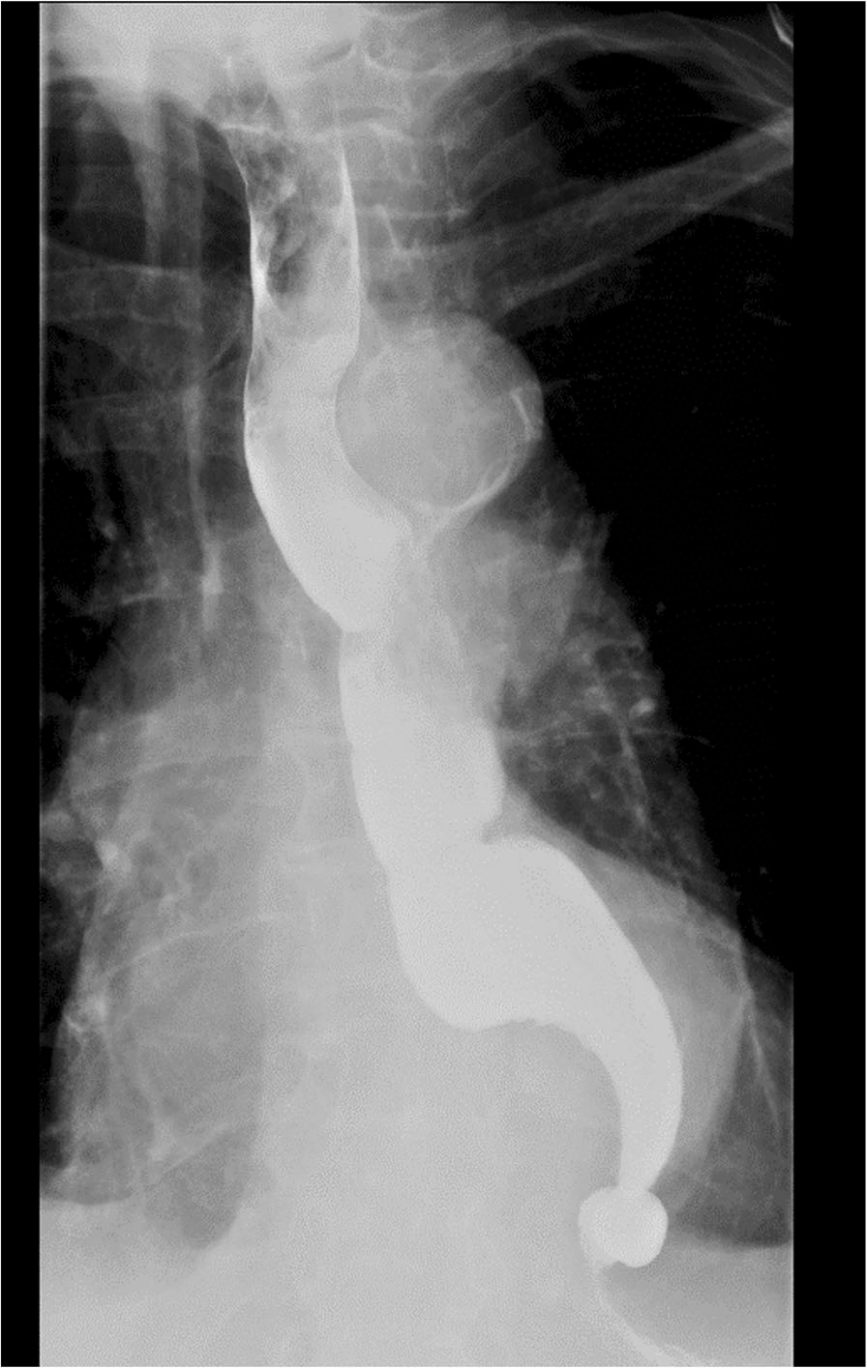
Barium swallow showing a ‘bird’s beak’ sign at the distal esophagus

A 84-year-old Caucasian woman presented to our emergency department after 7 weeks of severe anorexia and unintentional weight loss of 8 kg over the last 3 months to 47.7 kg. She complained of bad taste and dysphagia to solids, but not to liquids. Furthermore she mentioned regurgitation of undigested food. Clinical examination was unremarkable except of the observed weight loss. Her medical history included arterial hypertension, hyperlipidaemia, COPD, chronic atrial flutter, heart failure, previous lung embolism, radioactive iodine therapy for hyperthyroidism and previous drainage of a subdural hematoma of the thoracolumbar spine. Blood analysis showed a digitalis intoxication, a slightly elevated C-reactive protein without leucocytosis and discrete hypoalbuminemia and a grade 2 chronic kidney disease (CKD-epi). Thyroid function was normal. Her chest radiograph showed a widened mediastinum with aortic unfolding and calcification. Since anorexia might be caused by digitalis, this therapy was discontinued. However, without alleviation of the symptoms. To exclude a Zenker’s diverticulum, a barium swallow evaluation was performed, showing mild dilatation of the proximal esophagus with distal stasis of food and marked hypocontractility, presenting as a ‘bird’s beak sign’ (Fig. 1). Endoscopic evaluation was normal, without food remnants or evidence of esophageal neoplasm (fig. S1 A-B). The latter is important to consider in elderly patients with dysphagia to solids. During the procedure a nasogastric tube was placed for supplemental feeding and the patient gained 4 kg.

**Fig. 2:**
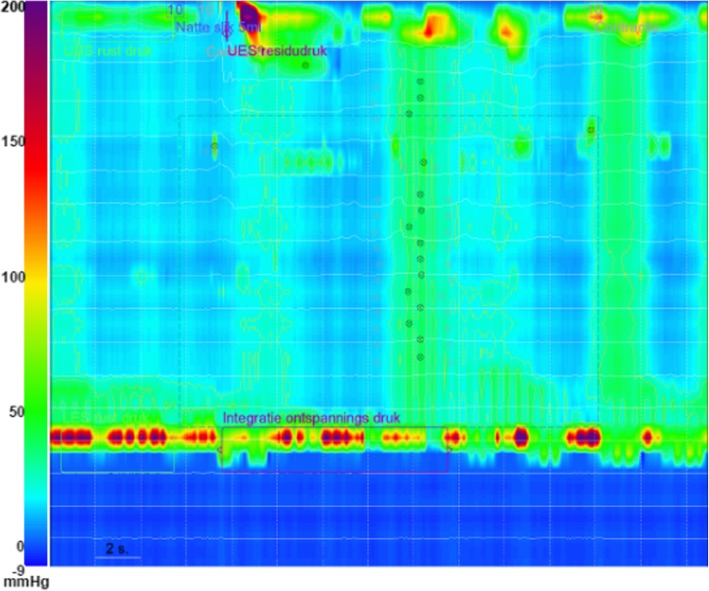
High resolution manometry showing panesophageal pressure rise

Subsequent manometric examination displayed insufficient relaxation of the LES (IRP4 75 mmHg, normal < 20 mmHg) combined with a mean LES resting pressure of 156 mmHg (normal 10-35 mmHg) and a panesophageal pressure rise of > 30 mmHg in more than 20% of swallows, compatible with a type 2 achalasia according to the Chicago classification for esophageal motility disorders (Fig. 2) [3].

**Fig. 3:**
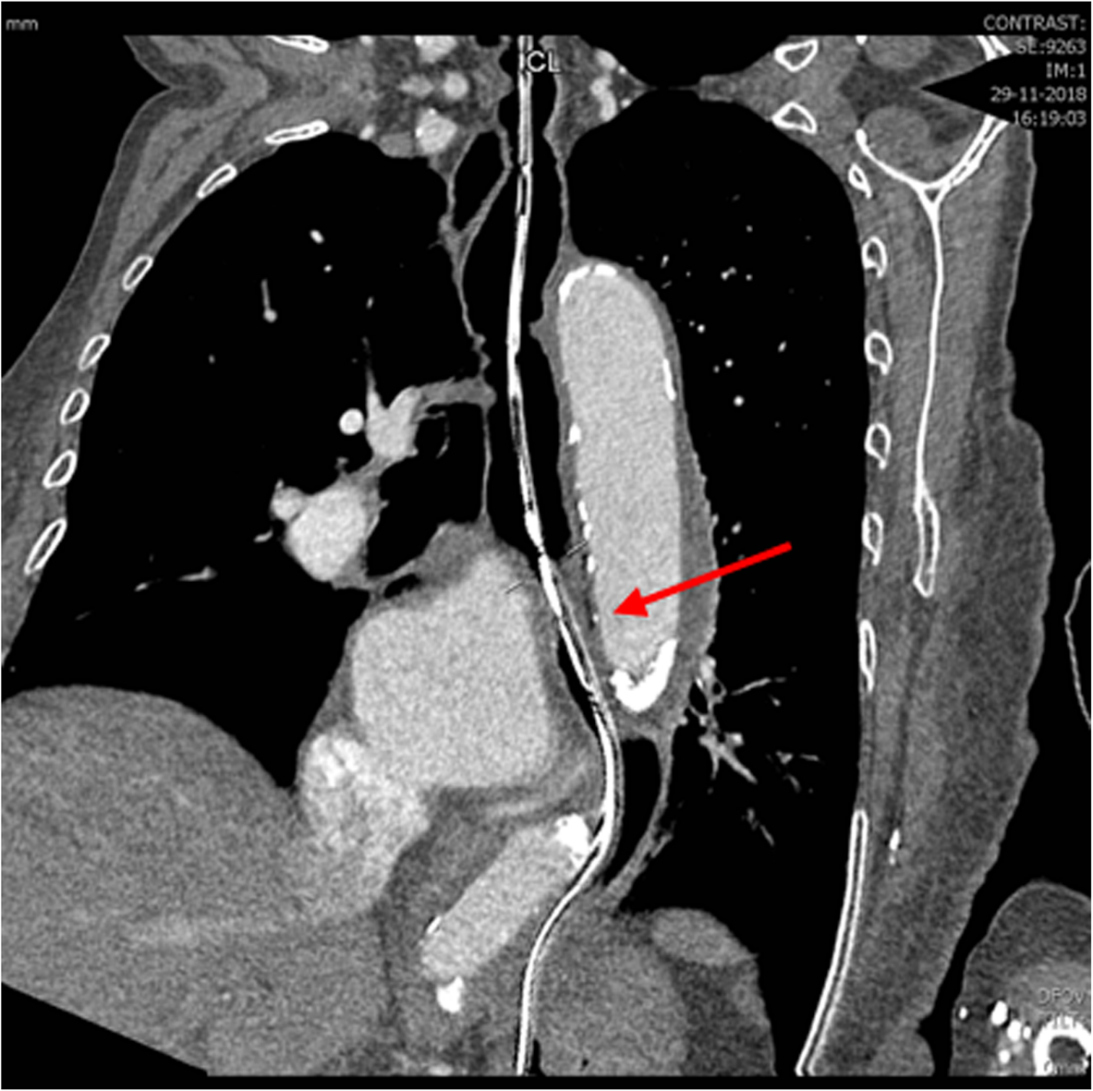
Coronal CT reconstructed image on esophagus; arrow indicates compression of distal esophagus (with nasogastric tube in situ) by aortic aneurysm

Since type 2 achalasia is associated with external compression of the esophagus a computed tomographic (CT) evaluation of the chest was performed to exclude pseudoachalasia, revealing compression of the distal esophagus by a dissecting aorta descendens, type Stanford B (40 mm) (Fig. 3, fig. S2–S3). The patient was not considered suitable for vascular repair given the multiple comorbidities and she was managed with a liquidised diet and discharged home. Her blood pressure was already well controlled with antihypertensive medication.

Six weeks later, the patient was readmitted. Since she was not able to maintain a stable body weight and had lost another 6 kg, she was progressively started on nasogastric tube feeding, with close biochemical monitoring to prevent refeeding syndrome. In consent with the patient, a percutaneous endoscopic gastrostomy (PEG) was placed 2 weeks later and enteral feeding was continued. Unfortunately another 2 weeks later the patient developed a pulmonary infection complicated with severe cardiac decompensation and inevitably passed away.

## Discussion and conclusion

Pseudoachalasia was first described by Ogilvie in 1947 as a secondary form of achalasia [5]. Achalasia is an uncommon esophageal motor disorder (1 in 100,000 individuals) caused by loss of inhibitory neurons in the esophageal wall. Pseudoachalasia is seen in up to 4.7% of patients with symptoms and esophageal manometric findings mimicking primary achalasia [6, 7]. A literature review on pseudoachalasia reported an underlying malignant cause in more than 70%, and a relation to previous surgical procedures in 12% of the cases [7].

Patients with pseudoachalasia often present with a more recent onset of symptoms than those with achalasia – typically first to solids and with worsening of the condition, additionally to liquids. Constitutional symptoms such as anorexia, unintentional weight loss and malnutrition develop during course of the disease. Classically, laboratory findings are completely normal except for markers of a catabolic state such as hypoalbuminemia. The work-up includes endoscopic evaluation to exclude an underlying malignant process, followed by a high resolution manometry and barium swallow test. Even if all investigations are consistent with achalasia, computed tomography of the thorax or endoscopic ultrasonography should be performed in the aged patient (> 60 year) with substantial weight loss and short duration of symptoms (< 1 year) to rule out other causes and exclude pseudoachalasia.

To our knowledge, only one previous case report described the pitfall of missing pseudoachalasia secondary to vascular compression when the initial examinations indicate primary achalasia [8]. Dysphagia caused by an aneurysm of the aorta is called dysphagia aortica. In this rare condition the aorta extrinsically compresses the esophagus against the left atrium, thereby mechanically obstructing the food bolus to be propulsed distally. Dysphagia aortica is typically associated with female gender, old age, hypertension, short stature, and kyphoscoliosis [9, 10].

Our patient was not suited for surgical intervention and for mild cases of dysphagia aortica conservative measures, such as diet modifications, nutritional supplements, and use of prokinetics have demonstrated to be beneficial. When confronted with more severe symptoms, surgical correction of the aneurysm or dissection should be considered. In patients who are not deemed fit for surgery, enteral nutrition through nasogastric tube or PEG placement might be an option [11]. It is clear from this case that it is important to differentiate pseudoachalasia from achalasia in elderly patients where primary investigations are suggestive for primary achalasia. Additional imaging should be performed to rule out secondary causes of achalasia.

## Supplementary information

**Additional file 1 : Figure S1.** A-B: Endoscopic view on distal esophagus showing the Z-line. Arrow indicates nasogastric tube in panel A.

**Additional file 2 : Figure S2.** Sagittal CT image depicting the compression of distal esophagus by aortic aneurysm. Arrow indicates nasogastric tube.

**Additional file 3 : Figure S3.** Cross-sectional CT image at the diaphragm level showing compression of distal esophagus by aortic aneurysm. Arrow indicates nasogastric tube.

## Data Availability

This case report contains clinical data from the electronic medical record in the Leuven University Hospitals. Additional information is available upon request, but only in accordance with the privacy restrictions of the hospital.

## Abbreviations

cm: centimeters
COPD: Chronic obstructive pulmonary disease
CT: Computer tomography
kg: kilograms
LES: Lower esophageal sfincter
mm: millimeters
mmHg: millimeter of mercury
PEG: Percutaneous endoscopic gastrostomy
