# Supplementary figures and images for: Case report: an aortic aneurysm as cause of pseudoachalasia
Source: BMC Gastroenterol. 2020 Mar 6;20:63. doi: 10.1186/s12876-020-01198-y (PMC7060513; doi:10.1186/s12876-020-01198-y)

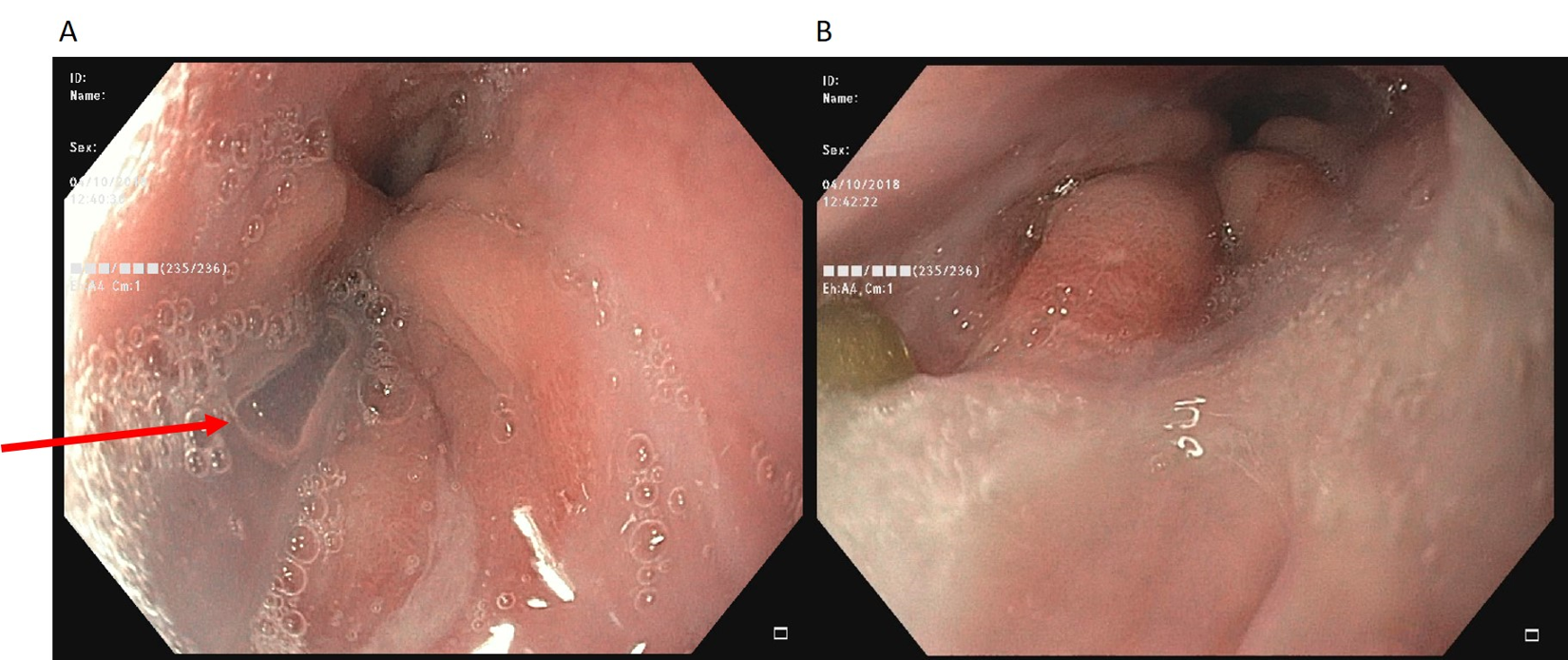

Supplement: Supplementary file 1 — Additional file 1 : Figure S1. A-B: Endoscopic view on distal esophagus showing the Z-line. Arrow indicates nasogastric tube in panel A. [file 12876_2020_1198_MOESM1_ESM.png]

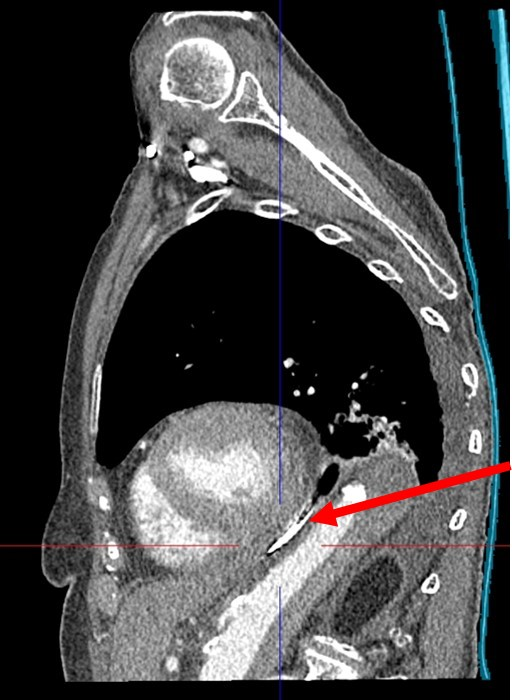

Supplement: Supplementary file 2 — Additional file 2 : Figure S2. Sagittal CT image depicting the compression of distal esophagus by aortic aneurysm. Arrow indicates nasogastric tube. [file 12876_2020_1198_MOESM2_ESM.png]

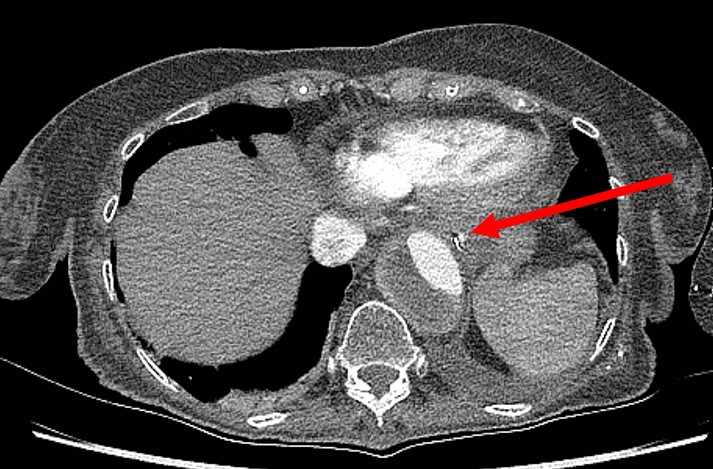

Supplement: Supplementary file 3 — Additional file 3 : Figure S3. Cross-sectional CT image at the diaphragm level showing compression of distal esophagus by aortic aneurysm. Arrow indicates nasogastric tube. [file 12876_2020_1198_MOESM3_ESM.png]
